# Cancer stem-like cells stay in a plastic state ready for tumor evolution

**DOI:** 10.1016/j.neo.2025.101134

**Published:** 2025-02-06

**Authors:** Jiali Xu, Houde Zhang, Zhihao Nie, Wenyou He, Yichao Zhao, Zhenhui Huang, Lin Jia, Zhiye Du, Baotong Zhang, Siyuan Xia

**Affiliations:** a Department of Human Cell Biology and Genetics, School of Medicine, Southern University of Science and Technology, Shenzhen 518055, China; b Coulter Department of Biomedical Engineering, Georgia Institute of Technology and Emory University, Atlanta, GA, USA; c College of Pharmacy, Shenzhen Technology University, Shenzhen 518118, Guangdong, China; d Musculoskeletal Tumor Center, Peking University People's Hospital, Beijing, China

**Keywords:** Cell plasticity, Cancer stem-like cell (CSC), Tumor heterogeneity, Therapeutic resistance, Cancer metastasis, Chemotherapy, Solid tumor

## Abstract

Cell plasticity emerges as a novel cancer hallmark and is pivotal in driving tumor heterogeneity and adaptive resistance to different therapies. Cancer stem-like cells (CSCs) are considered the root of cancer. While first defined as tumor-initiating cells with the potential to develop a heterogeneous tumor, CSCs further demonstrate their roles in cancer metastasis and adaptive therapeutic resistance. Generally, CSCs come from the malignant transformation of somatic stem cells or the de-differentiation of other cancer cells. The resultant cells gain more plasticity and are ready to differentiate into different cell states, enabling them to adapt to therapies and metastatic ecosystems. Therefore, CSCs are likely the nature of tumor cells that gain cell plasticity. However, the phenotypic plasticity of CSCs has never been systematically discussed. Here, we review the distinct intrinsic signaling pathways and unique microenvironmental niches that endow CSC plasticity in solid tumors to adapt to stressful conditions, as well as emerging opportunities for CSC-targeted therapy.

## Highlights

• The plasticity of CSCs results in intratumor heterogeneity in solid tumors.
• CSCs play a central role in the development of adaptive therapeutic resistance.
• CSC markers and intrinsic signaling pathways inspire CSC-targeted therapies.
• Unique CSC microenvironment provides valuable insights for CSC-targeted therapies.

## Introduction

In 1994, John Dick made the significant discovery that not all cancer cells follow the same developmental pathway. He observed that only a small subset of leukemia cells possessed self-renewal and tumor-generating abilities, while most other cancer cells exhibited limited or no capacity to promote tumor growth. To describe these mutated cells with tumor-initiating potential, John Dick coined the term "cancer stem-like cells" (CSCs) [4]. Another notable example of CSCs emerged that same year in a seminal study on acute myeloid leukemia (AML) by Lapidot et al. The study identified a subset of poorly differentiated CD34+/CD38- cells with stem cell-like self-renewal ability and tumor-initiating potential. Transplantation of these cells into severe combined immunodeficiency (SCID) mice led to the development of human AML [47]. Subsequent research has provided accumulating evidence supporting the existence of CSCs in various solid tumors, including but not limited to the brain [32,100], prostate [13,127], colon [53,79], pancreas [50], ovary [128], and lung [17,23].

Traditional tumor therapy includes surgical resection, radiotherapy, and chemotherapy, which largely improve patient survival, but the subsequent tumor metastasis and adaptive drug resistance make the treatment outcome unsatisfactory. While targeted therapy and immunotherapy have further improved the survival rates among cancer patients, therapeutic resistance is still a challenge due to the presence of tumor heterogeneity and immunosuppressive tumor microenvironment. Many clinical observations initially indicate that cancer cells seem to have been eradicated, but then they often relapse. The containment of CSCs is a pivotal cell-level mechanism by which tumors maintain their heterogeneity and acquire the capability of therapeutic resistance, recurrence, and metastasis, rendering cancer an incurable disease.

## Main markers of cancer stem-like cells

Identifying and isolating CSCs provides valuable information for developing targeted therapies. A surface marker panel has been identified for solid CSCs, such as CD24, CD44, CD54, CD117, CD133, etc. (Table 1). Certain markers play significant roles in molecular signaling transduction, extending beyond their function as mere indicators of CSCs. One example of such markers is ALDH, as discussed in our previous review [62].

**Table 1:** Cancer stem-like cell marker in solid tumors.

**Markers**	**Cancer types**	**Contribution**	**Pathway**	**Reference**
CD44	Head and Neck Squamous Cell Cancer	Tumorigenicity	Increases PI3K-4EBP1-SOX2 signaling and promotes invasive signaling by Ezrin/PI3K	[27,85]
	Prostate Cancer	Tumor proliferation, drug resistance	CD44+/α2β1hi/CD133+ cells differentiate into an AR-positive phenotype similar to prostate cancer in situ, becoming resistant to androgen deprivation therapy	[13]
	Pancreatic Cancer	Tumorigenicity	Promotes cancer stemness via SPP1-CD44 axis	[50,72]
	Ovarian Cancer	Cell proliferation, differentiation, motility, chemoresistance	Stimulates EGFR-Ras-ERK as an HA receptor	[11,128]
CD24	Head and Neck Squamous Cell Cancer	Tumorigenicity	-	[85]
	Pancreatic Cancer	Tumorigenicity	-	[50]
CD133	Brain Cancer	Chemotherapy, tumorigenesis, cell differentiation, and migration	Recruits HDAC6 to deacetylate β-catenin for its stabilization; Activates the PI3K/Akt pathway and promotes tumorigenesis of glioma stem cells; Interacts with Src to activate FAK	[32,100,117]
	Prostate Cancer	Tumor proliferation, drug resistance	CD44+/α2β1hi/CD133+ cells differentiate into an AR-positive phenotype similar to prostate cancer in situ, becoming resistant to ADT	[13]
	Colon Cancer	Initiation and maintenance of tumor growth	Activates PI3K/AKT signaling	[75,79,86]
	Lung Cancer	Tumorigenicity	Interacts with HK2 to inhibit self-ubiquitination and interpretation	[23,112]
CD54	Prostate Cancer	Self-renewal and tumorigenicity	Mediates CSC regulation via the p38-Notch1 axis	[13,51]
CD117	Ovarian Cancer	Regulation of cell proliferation, apoptosis, and adhesion by binding to its cytokine ligands	Activates PI3K/Akt, Ras/ERK, Src and JAK/STAT pathways	[103]

CD44 has been identified as a marker for CSC identification in head and neck, prostate, ovarian, and pancreatic cancer. In head and neck cancer, CD44 enhances the PI3K-4EBP1-SOX2 signaling pathway and promotes invasive signaling through Ezrin/PI3 K [27]. CD44 promotes cancer stemness in pancreatic cancer through the SPP1-CD44 axis [72]. In prostate cancer, CD44+/α2β1^hi^/CD133+ cells undergo cell differentiation to an androgen receptor-positive phenotype similar to prostate cancer in situ [13]. In ovarian cancer, CD44 is a hyaluronic acid receptor and stimulates the EGFR-Ras-ERK pathway [11].

CD133 is another surface marker that plays a key role in signaling transduction. For example, CD133 participates in signaling pathways such as β-catenin, PI3K/Akt, and Src-FAK in brain cancer and promotes tumorigenesis and migration [32,100,117]. CD133 activates the PI3K/AKT signaling pathway to maintain colon cancer [79]. In addition, CD133 interacts with HK2 in lung cancer to inhibit its ubiquitination and degradation [112].

In addition to CD44 and CD133, which have been extensively studied, other markers have also been identified in solid CSCs. CD24 has been implicated in promoting tumorigenesis in head and neck cancer [85] and pancreatic cancer [50], although the specific underlying pathways are still unknown. CD54 has been identified in prostate cancer and regulates CSCs through the p38-Notch1 axis [51]. Furthermore, CD117 regulates PI3K/Akt, Ras/ERK, Src, and JAK/STAT, and CD117-positive cells have been found to exhibit higher tumorigenicity and resistance in ovarian cancer, making it a potential marker for ovarian CSCs [60].

## The plasticity of CSCs results in intratumor heterogeneity and therapeutic resistance in solid tumors

CSCs are a small cell population with unlimited proliferative potential, contributing to tumorigenesis. They maintain a relatively stable proportion within tumors through self-renewal and differentiation. The clonal evolution and differentiation of CSCs results in tumor heterogeneity, a leading cause of therapeutic resistance. The genetic alterations and intrinsic properties of CSCs contribute to treatment resistance [67], and both internal and external factors regulate the biological identities of CSCs. The internal regulators encompass genetic, epigenetic, and metabolic regulations, while the external regulators include niche factors, the immune system, and interactions with the tumor microenvironment (TME) [49].

Compared to other cancer cells that divide rapidly around CSC subsets, CSCs are usually more resistant to currently available anticancer therapies. One of the reasons is that CSCs are usually in a relatively static cell cycle. CSCs are insensitive to radiotherapy and chemotherapy drugs, and they can re-enter the cell cycle under the action of some stimulators, resulting in rapid proliferation, drug resistance, and recurrence of tumors [96]. The plasticity of CSCs enables them to rapidly adapt to the changes caused by treatment and TME in the whole process of tumor evolution, which is conducive to advanced tumor progression [1,34,83]. Moreover, CSCs alter the signal transduction molecules that control drug metabolism. For example, CSCs increase the drug efflux rate and the high expression of ATP-binding cassette transporters [28].

CSCs promote tumor metastasis through distal dissemination, which is facilitated by mechanisms such as epithelial-mesenchymal transition (EMT) and immune escape [111]. During EMT, cancer cells in an epithelial state undergo a phenotypic transformation into mesenchymal-like cells. Mesenchymal cells lose cell polarity, decrease intercellular adhesion, and enhance their migratory and invasive capabilities. Mesenchymal cells show downregulation of epithelial markers such as E-cadherin and keratin, and upregulation of mesenchymal markers including vimentin, fibronectin, N-cadherin, and α-SMA. EMT is well recognized for its crucial role in tumor metastasis [25]. Accumulating evidence suggests that EMT is a plastic process by which non-stem cancer cells acquire CSC features, such as the scenarios in breast cancer [16,65] and bladder cancer [129]. Interestingly, CSCs also have their own heterogeneity and can switch between mesenchymal and epithelial phenotypes. For instance, CD24-/CD44+ breast CSCs are mesenchymal-like, primarily quiescent, and localized at the tumor-invasive front, whereas ALDH+ breast CSCs are epithelial-like and proliferative, and more centrally located [56].

## Mechanism of therapeutic resistance mediated by intrinsic signaling in CSCs

**Fig. 1:**
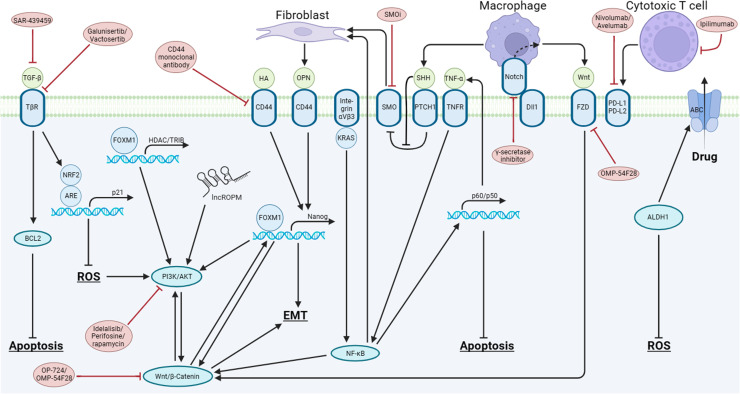
**Intrinsically activated signaling pathways in CSCs contribute to therapeutic resistance in solid tumors.** In CSCs, the crosstalk between the β-catenin/Wnt and PI3K/AKT pathways promotes EMT and contributes to therapeutic resistance. NF-κB, activated by integrin αVβ3 and TNF-α, sustains the activation of the β-catenin/Wnt pathway and enhances the anti-apoptotic pathways. The Notch pathway, activated by macrophages in TME, triggers the activation of the β-catenin/Wnt pathway. Hedgehog signaling, regulated by macrophages, stimulates fibroblasts to secrete osteopontin (OPN), which binds with CD44 to promote the transcription of Nanog, contributing to EMT. TGF-β inhibits ROS levels and apoptotic pathways. CTLs interact with CSCs by PDL1/2 pathways. Strategies for intervening CSC pathways are illustrated in the figures by red ellipse. This figure is generated by Biorender with an agreement number of VQ26395CIC.

CSCs divide more slowly and have robust DNA repair mechanisms compared to other non-CSCs in the bulk of solid tumors [97]. The unique features of CSCs render them less responsive to therapies and more challenging to eradicate. For instance, residual CSCs give rise to fast-proliferating cells after chemotherapy, leading to tumor recurrence. Accumulating studies have unveiled a distinct map of signaling pathways within the CSCs of solid tumors. Herein, we systematically review these pathways and aim to provide insights into potential therapeutic strategies (Fig. 1 and Table 2).

**Table 2:** Intrinsic pathways within CSCs cause chemoresistance.

**Pathway**	**Cancer types**	**Tolerant therapies**	**Mechanism of resistance**	**Reference**
Notch	Lung cancer	Erlotinib	Activated Notch activity	[52]
	Breast cancer	-	Increased expression of Wnt family ligands	[9]
Wnt/β-catenin	Ovarian cancer, breast cancer, glioblastoma, colorectal cancer	Tamoxifen, doxorubicin, cisplatin	Induce multidrug resistance–associated protein-1(MRP-1) expression	[35,58,61,64,96]
PI3K/AKT/mTOR	Glioblastoma	Temozolomide	Activate Wnt/β-catenin pathway	[107]
	Colorectal cancer, Small cell lung cancer	5-fuorouracil	Upregulate HIF-1α	[20]
	Prostate cancer	Docetaxel	More P-gp expression	[59]
	Breast cancer	Palbociclib	More phosphorylated 4E-BP1 and higher levels of Cyclin D1 and CDK4 translation.	[7]
	NSCLC	Sotorasib	Activate PAK and RAS	[10]
NF-κB	Glioblastoma	Doxorubicin	Upregulate expression of MDR1	[15,108,120]
	Breast cancer	Paclitaxel	Produce IL-6, IL-8, CXCLs	[115]
	Ovarian cancer	Cisplatin	Drive CSCs into an undifferentiated state with anti-apoptotic properties	[24,105]
Hedgehog	Hepatocellular carcinoma	sorafenib	TSPAN8 inhibits the degradation of the SHH/ PTCH1 complex and results in the translocation of SMO to cilia	[114,130]
TGF-β	Squamous cell carcinoma	Cisplatin	Aactivates p21	[3,14,96]
	Prostate cancer	Docetaxel	Protect Bcl-2 from degradation	[54,125]
CD55	Endometrioid tumors	cisplatin	Regulated self-renewal and core pluripotency genes via ROR2/JNK signaling	[24,92]
Nanog	Ovarian cancer	Doxorubicin and paclitaxel	Activate EMT, β-catenin accumulation in the nucleus and Wnt signaling	[5,60,96]
Aldehyde dehydrogenase (ALDH)	Colonic adenocarcinoma and ovarian cancer cells	Oxazaphosphorine (OP) family, capecitabine, 5-FU, taxane and platinum	Eliminate oxidative stress	[2,24,43,99,124]
ATP-binding box (ABC) transporters	Leukemias and some solid tumors	Vinca alkaloids, the anthracyclines, the RNA transcription inhibitor actinomycin-D and the microtubule-stabilizing drug paclitaxel	Expel drugs	[28,124]
Integrin αvβ3	Breast, lung and pancreatic carcinomas	Erlotinib	Recruit KRAS and RalB to the tumor plasma membrane, leading to the activation of TBK1 and NF-κB	[30,94]
FOXM1	Pancreatic cancer	Cisplatin	CBP and ß-catenin/FOXM1 transcription complex drive gene expression	[12,44,96]
	Breast cancer	Trastuzumab	GDF-15 stimulates the phosphorylation of p38	[21,69,96]
TRIB1	Non-small cell lung cancer	Cisplatin	Activate MEK-ERK pathway, GRP78-Akt pathway, and C/EBP	[24,113]
lncROPM	Breast carcinomas	Tamoxifen, doxorubicin, cisplatin	Activate PI3K/AKT, Wnt/β-catenin, and Hippo/YAP signaling	[57]

### Notch

Notch signaling is activated in CSCs through various mechanisms, all of which lead to therapeutic resistance. For instance, Erlotinib-resistant PC9 cells (PC9ER) and Erlotinib-resistant HCC827 cells (HCC827ER) both exhibit partial stemness characteristics. The miR-146a/Notch signal remains highly activated in an m6A-dependent manner in these drug-resistant cells. LncRNA TUSC7 can suppress the Notch signaling, rendering the cells sensitive to Erlotinib. The high level of m6A reader YTHDF2 inhibits TUSC7 in CSCs, ultimately leading to chemotherapy resistance [52]. Interestingly, the activation of Notch signaling in CSCs is crucial for their interaction with macrophages. Mammary stem cells (MSCs) are enriched for the Notch pathway ligand Dll1. Notch signaling mediated by Dll1 maintains stemness by activating Notch signaling in macrophages, resulting in increased expression of Wnt family ligands (such as Wnt3, Wnt10A, and Wnt16). Wnts take turns maintaining the stemness of MSCs [9].

### β-catenin/Wnt

The activation of the β-catenin/Wnt pathway in CSCs is another factor contributing to therapeutic resistance. In glioblastoma, Wnt activation leads to the upregulation of multidrug resistance-associated protein 1 (MRP-1) expression, ultimately causing the acquisition of stem cell-like properties and resistance to chemotherapy [35]. In colorectal cancer (CRC), METTL3-mediated m6A modification enhances the binding of Sec62 with β-catenin, thereby enhancing Wnt signal transduction, promoting CRC stemness, and augmenting chemotherapy resistance [58]. A high number of CSCs have been identified in triple-negative breast cancer, where they are driven by the β-catenin/FOXM1 transcription complex, affecting tumor resistance [96]. Therefore, an association has been established between FOXM1 and ovarian cancer stemness, with overexpression of FOXM1 in cisplatin-sensitive ovarian cancer cell lines promoting drug resistance [12,96].

### PI3K/AKT

The PI3K/AKT pathway itself plays a significant role in chemotherapy resistance in CSCs. Analysis of transcriptome data from public databases confirmed the upregulation of the PI3K/AKT and HIF-1 pathways in chemotherapy-resistant SCLC cell lines [37]. In the context of 5-FU resistance in CRC, the upregulation of HIF-1α activates PI3K/AKT signaling, which is mediated by reactive oxygen species (ROS) and abnormal activation of β-catenin [20]. Taxane-resistant subclones display increased activation of the androgen receptor and the PI3K/AKT pathway in comparison to parental cells. Additionally, they exhibit a more distinct mesenchymal and stem cell-like phenotype, accompanied by an increase in P-glycoprotein (P-gp) expression [59]. Furthermore, the PI3K/mTOR pathway becomes excessively activated in palbociclib-resistant cells, leading to increased phosphorylation of 4E-BP1 and higher translational levels of cyclin D1 and CDK4. P21-activated kinases (PAKs) are activated in resistant cells, and they phosphorylate MEK at threonine 298 and c-Raf at serine 338, thereby activating the mitogen-activated protein kinase pathway [7]. Additionally, the PI3K/AKT pathway displays constitutive activity in sotorasib-resistant cells. Moreover, the PI3 K and PAK pathways form a mutually reinforcing regulatory loop, mediating resistance to sotorasib [10]. Glioblastoma stem-like cells (GCSCs) significantly promote drug resistance and demonstrate enhanced DNA damage repair capabilities. The expression of CD133 in these cells, along with DNA-PK, possibly contributes to increased MDR3 through the PI3K/AKT signaling pathway. Additionally, downstream targets of PI3 K, Akt, and nuclear factor (NF)-κB, which interacts with the MDR1 promoter, are elevated in these cells [120]. The enrichment of CSCs triggered by cisplatin treatment activates the PI3K/AKT pathway by increasing the activity of the TRIB oncogene and class I histone deacetylases (HDACs) [113].

### NF-κB

NF-κB activation by doxorubicin has been observed to suppress cell apoptosis [109]. In its unliganded state, the αvβ3 integrin recruits KRAS and RalB to the cell membrane, activating TANK-binding kinase 1 (TBK-1) and NF-κB [94]. Overexpression of IRAK1 confers a growth advantage to triple-negative breast cancer (TNBC) cells by enhancing the secretion of NF-κB-related cytokines. Treatment with paclitaxel induces robust phosphorylation of IRAK1, the enrichment of CSCs, and the development of acquired resistance to paclitaxel treatment [116]. The activation of NF-κB/TNF-α /PIK3CA signaling pathway increases CSCs, causing resistance to cisplatin in ovarian cancer [105]. Conversely, the administration of an altered version of IκBα, an inhibitor of NF-κB, suppresses NF-κB activity. This process renders chemoresistant tumors susceptible to apoptosis triggered by tumor necrosis factor κ (TNFκ) and the chemotherapeutic agent CPT-11 [108].

### Hedgehog

In breast CSCs, TSPAN8 interacts with the Hedgehog receptor PTCH1 and inhibits the degradation of the SHH/PTCH1 complex by recruiting the deubiquitinase ATXN3. This leads to the translocation of SMO to cilia and chemoresistance in CSCs [130]. CD44-positive hepatocellular carcinoma (HCC) patient-derived organoids (PDOs) exhibit significant resistance to sorafenib, and sorafenib increases CD44 levels. Drug screening shows that compared to Notch, Hippo, and Wnt signaling inhibitors, Hedgehog pathway suppression by GANT61 effectively inhibits the viability of HCC PDO cells [114].

### Transforming growth factor-β (TGF-β)

TGF-β plays a dual role in tumorigenesis. In the initial phases of tumor development, TGF-β is a tumor suppressor by promoting cell cycle arrest and apoptosis in cancer cells. However, TGF-β promotes drug resistance as tumors progress by activating specific transcription factors. For example, TGF-β promotes heterogeneity and drug resistance in squamous cell carcinoma by stabilizing NRF2, transcriptionally activating p21, significantly enhancing glutathione metabolism, thus reducing ROS levels and consequently diminishing the effectiveness of anticancer treatments [76]. Additionally, in prostate cancer, acetylation of lysine 369 of KLF5 mediates docetaxel resistance both in vitro and in vivo. The TGF-β/acetylated KLF5 signaling axis can transcriptionally activate Bcl-2 expression. DTX-induced degradation of Bcl-2 relies on the proteasomal pathway, but TGF-β inhibits docetaxel-induced Bcl-2 ubiquitination [54,125].

### CSC markers

Surface markers of CSCs serve as identifiers for CSCs and actively participate in signaling transduction processes. For instance, CD55, CD44, CD133 and CD117 in CSCs play their roles in chemoresistance. CD55 was found to be highly expressed in CSCs in endometrioid tumors, and it can induce DNA repair through ROR2/JNK and lymphocyte-specific protein tyrosine kinase (LCK) signaling, leading to cisplatin resistance [92]. CD133 is essential for CSCs in ovarian cancer and glioblastoma, affecting their response to chemotherapy [60]. CD44+/CD24-/low mammospheres are the main component of residual cells after docetaxel treatment and can lead to tumor recurrence in breast cancer [14]. Moreover, CSCs coexpressing CD117 and CD44 exhibit chemoresistance in ovarian cancer [60].

CD44 interacts with hyaluronic acid (HA) and initiates various signaling pathways. CD44 signaling activates NANOG and its downstream target genes, leading to chemoresistance [60]. The expression level of NANOG has also been found to increase in cell lines with high resistance to cisplatin [89]. In gastric cancer, NANOGP8, a functional paralog of NANOG, acts as a key regulator of CSCs. NANOGP8 promotes EMT by inducing the nuclear accumulation of β-catenin and the activation of the Wnt signaling pathway [24,63].

Aldehyde dehydrogenase (ALDH), another canonical CSC marker in different types of solid tumors, has been shown to be associated with tumor drug resistance [60]. The direct pathways modulated by ALDH have been reviewed previously [62]. Generally, retinoic acid metabolism regulated by ALDH plays a pivotal role in the signaling pathways downstream of ALDH. ALDH1A1 can eliminate oxidative stress and reduce sensitivity to oxazolidine, taxane, and platinum-based drugs [99,124]. ALDH1A1 also plays a promoting role in oxazepathine (OP) family resistance in colorectal cancer cells [43]. In addition, ALDH1A1 can regulate adenosine triphosphate binding box (ABC) drug transporters, increasing ovarian cancer resistance to taxane and platinum [60].

ABC transporters, such as MDR1 (ABCB1), MRP1 (ABCC1), and ABCG2, are prominently expressed in CSCs. These transporters protect cells from the accumulation of toxic compounds, thereby expelling drugs and reducing cell sensitivity to chemotherapeutic agents [2,28,124].

Integrin αvβ3 is a marker for CSCs in breast, lung, and pancreatic cancers and exhibits high resistance to receptor tyrosine kinase inhibitors like Erlotinib. Mechanistically, in its unliganded state, αvβ3 can recruit KRAS and RalB to the tumor plasma membrane, activating TBK1 and NF-κB signaling pathways [30,93].

### LncROPM

LncROPM is a lncRNA with abundant expression in breast CSCs. LncROPM has been found to promote phospholipid metabolism and free fatty acid production in BCSCs. This, in turn, activates signaling pathways such as PI3K/AKT, Wnt/β-catenin, and Hippo/YAP. The activation of these pathways results in drug resistance [57].

## Unique crosstalk between CSCs and TME maintains stemness

TME serves a pivotal role in tumor growth and metastasis. Notably, the evolution of tumor cells is accompanied by TME evolution. Simultaneously, TME significantly influences tumor resistance to therapies. Macrophages, fibroblasts, and cytotoxic T cells primarily engage with CSCs in the TME through paracrine factors and other mechanisms, consequently impacting the therapeutic resistance of CSCs (Table 3).

**Table 3:** Tumor microenvironment interacts with CSCs and causes chemoresistance.

**TME**	**Cancer types**	**Tolerant drugs**	**Mechanism of resistance**	**Reference**
Macrophage	Advanced hepatocellular carcinoma	Sorafenib	M2 TAMs release CXCL1 and CXCL2 and affect BCL-2 family gene expression	[110]
	Colon cancer	Small-molecule SMO inhibitors (SMOi)	TAMs produce MFG-E8 and IL-6	[101,118]
	Breast cancer	Paclitaxel and tamoxifen	Interleukin 10 (IL-10) activates STAT3/Bcl-2	[123]
	Breast cancer	CDDP	Associate with the EGFR/STAT3/SOX2	[38,87]
	Breast cancer	etoposide (Etoposide)	Induce Wnt-1 upregulation which downregulates E-cadherin junctions in the HER2+ cancer cells	[55]
Fibroblast	Breast and lung cancer	Docetaxel or cisplatin	CD10+ GPR77+ CAFs are driven by NF-kB activation via p65 phosphorylation and acetylation	[102]
	Pancreatic cancer	Docetaxel	Long-term treatment of PC cells with CAF-CM enriched stemness	[72,102]
	Triple negative breast cancer	Docetaxel	CAFs provide a supportive niche for CSC and express FGF5	[8]
Cytotoxic T cell	Multiple cancers	Immune therapy	CTL enriches a subset of cells with high expression of NANOG	[41]
	Multiple cancers	Immune therapy	CTL drives CSC-like phenotype through the Akt signaling pathway via transcriptional induction of Tcl1a by Nanog	[74]
	Small cell lung cancer	Immune therapy	T cells secret IFN-γ, inducing PD-L1 and PD-L2 expression in the CSC-like SCLC cells	[46]

### Macrophages

Tumor-associated macrophages (TAMs) signal CSCs through paracrine signaling and cause therapeutic resistance. For instance, M2 TAMs release paracrine factors, CXCL1 and CXCL2, which enhance resistance to sorafenib (SOR) in advanced-stage hepatocellular carcinoma (HCC) cells. These paracrine factors also promote the expression of BCL2 family genes, which diminishes SOR-induced apoptosis in CSCs [110]. IL6 signaling from macrophages in the tumor microenvironment can initiate the common HH-IL6 target gene promoter binding through GLI and STAT3 [101,118]. MFG-E8, predominantly generated by TAMs, initiates the Stat3 and Sonic Hedgehog signaling pathways in CSCs, thereby fortifying their resistance to chemotherapy [38,87]. Elevated levels of IL-10 secreted by TAMs contribute to chemoresistance in breast cancer by elevating BCL2 gene expression and upregulating STAT3 signaling [123]. CCL2, which is produced by cancer and bone marrow cells, attracts CD206+/Tie2+ macrophages and induces the upregulation of Wnt-1, leading to EMT in HER2+ breast cancer cells [55].

### Fibroblasts

Fibroblasts, a crucial component of the TME, also significantly impact the chemoresistance of CSCs. Specifically, CD10+ GPR77+ cancer-associated fibroblasts (CAFs) provide a supportive environment for CSC survival, promoting both tumor formation and chemoresistance. Mechanistically, the activation of NF-kB through sustained p65 phosphorylation and acetylation, driven by continuous complement signal transduction via GPR77 (a C5a receptor), fuels the activities of CD10+ GPR77+ CAFs [102]. Prolonged exposure of pancreatic cancer (PC) cells to CAF-derived media enhances stemness and notably upregulates OPN/SPP1 expression in PC cells [72]. Additionally, tumor cells release Hedgehog ligands, leading to the reprogramming of CAFs. This reprogramming not only creates a supportive niche for CSCs but also generates fibrous collagen, contributing to the development of chemotherapy-resistant CSC phenotypes through the expression of FGF5 [8]. In prostate cancer TME, an FGF9-TNFα crosstalk is established between CAFs and cancer cells for the resistance of AKT inhibitor capivasertib [126].

### Cytotoxic T cells

The secretion of IFN-γ by T cells induces high expression of PD-L1 and PD-L2 in CSC-like SCLC cells, promoting resistance to immune responses [46]. T cell-mediated immune selection drives regular tumor cells towards a CSC-like phenotype, and this transition to a CSC-like state is initiated through the Akt signaling pathway via Nanog transcription [74]. The immune pressure caused by cytotoxic T cells (CTLs) results in the enrichment of NANOG+ tumor cells with stem cell-like features and increased resistant to CTLs. This resistance is induced by LC3B through NANOG transcription, which leads to increased EGF secretion. Subsequently, overactivation of the EGFR-AKT signaling pathway confers resistance to T cell-mediated cytotoxicity in NANOG+ tumor cells [41]. The use of CTLs to target CSCs requires the elimination of CSCs by peptides that target antagonistic Notch and Numb proteins [68].

## Current therapeutic strategies for targeting CSCs

Given the distinct capabilities of CSCs in driving tumor relapse after treatment, including chemotherapies, there has been a growing focus on the development of therapeutic strategies that specifically target CSCs. These strategies are designed based on the unique surface markers, specific intrinsic signaling pathways, and the special microenvironmental interactions of CSCs. By targeting these aspects, researchers aim to develop treatments that can more effectively eliminate or inhibit CSCs, which could lead to more successful and long-lasting cancer therapies.

### Therapies based on specific surface markers of CSCs

CD44, CD44v4, and CD44v6 are three isoforms of CD44 commonly used as therapeutic targets. RO5429083 and SPL-108 are two CD44-targeting monoclonal antibodies undergoing clinical trials [26]. RO5429083 has been conducted among patients with CD44-expressing malignant solid tumors (NCT01358903). SPL-108 has been conducted with paclitaxel among patients with platinum-resistant CD44+ ovarian [26]. Results show that the overall response rate was 36 %, and all patients tolerated the maximum planned dose. AMC303, a peptide inhibitor of CD44v6 isoforms, is undergoing clinical Phase I/Ib trials as a monotherapy in patients with malignant solid tumors of epithelial origin (NCT number: NCT03009214) [66]. Salazosulfapyridine, an inhibitor of xCT, suppressed the proliferation of CD44v-positive cancer cells and finished clinical Phase I trials in combination with cisplatin and pemetrexed [77]. Overall response rate and median progression-free survival were 26.7 % and 11.7 months respectively, much better than that for cisplatin alone in previous studies.

AO-176 is a humanized anti-CD47 antibody that has completed phase I and II clinical trials to treat solid tumors [104]. Unlike other anti-CD47 antibodies, AO-176 has novel anticancer properties and negligible red blood cell binding side effects to avoid blood coagulation.

Catumaxomab, also called Removab, is a trifunctional bispecific antibody for both EpCAM and CD3 that has finished phase II and III trials (NCT number: NCT00836654) [31].

### Therapies targeting intrinsic pathways of CSCs

Although surface markers of CSCs provide a resource for the development of targeted therapy, inhibiting the intrinsic pathways are more likely to suppress the proliferation and maintenance of CSCs specifically and efficiently. JAK/STAT, PI3K-Akt-mTOR, TGF-β, and NF-κB-TNFα-PIK3CA are four pathways contributing to the proliferation of CSCs; Wnt, Notch, and Hedgehog are three pathways supporting CSC self-maintenance, stemness, and self-renewal.

In the strategies targeting the Wnt pathway, both β-catenin inhibitors (i.e., OP-724) and Wnt receptor inhibitors (i.e., OMP-54F28) have been developed for clinical trials. OP-724 is well tolerated by patients with advanced primary biliary cholangitis and shows antifibrotic effects [42]. Ipafricept (OMP-54F28) testing has been conducted among patients with pancreatic cancer, in combination with gemcitabine and paclitaxel in a Phase Ib Study [22]. Results show that 34.6 % of patients had a partial response, and 46.2 % had stable disease.

In the strategies targeting the Notch pathway, most drugs are γ-secretase inhibitors and have completed phase I trials, such as LY900009, MK0752, RO4929097, and LY3039478. Nirogacestat (PF-03084014) has completed phase III trials with frequent but low-grade adverse events [29,45]. LY900009, an oral Notch inhibitor targeting γ-secretase protein, has been tested among patients with advanced cancer, showing a high inhibition rate [78] and some dose-limiting toxicity. RO4929097, conducted on metastatic or locally advanced solid tumors [106], is also well tolerated. When conducted in combination with temsirolimus (Temsirolimus), RO4929097 exhibits significant efficiency and safety on advanced solid tumors [18].

In strategies targeting the Hedgehog (Hh) pathway, most Hh inhibitors specifically target Smoothened (SMO) and have been demonstrated to be effective in patients with basal cell carcinoma. The reason why Hh inhibitors show limited activity in other tumor types might be caused by crosstalk between Hh and other oncogenic signaling pathways or undiscovered Hh pathway activation mechanisms of CSCs in other tumor types. Vismodegib (GDC-0449) and glasdegib, the two most advanced Hh inhibitors, have entered phase II clinical trials. However, side effects should not be ignored [40,95]. A phase I study of vismodegib (GDC-0449) shows that around 25 % of patients were reported to experience serious adverse events, and even deaths due to adverse events were noted [95].

As for drugs that target the PI3K-Akt-mTOR pathway, Idelalisib (GS-1101), BKM120, and PX-866 have all entered phase II clinical trials, and the latter two trials have already been completed [33,36]. For PI3 K inhibitor BKM120 in the phase II clinical trial, pure BKM120 was associated with an unfavorable safety profile and minimal antitumor activity in monotherapy, and this trial was stopped before the end of recruitment for toxicity. For PI3 K inhibitor PX-866 in the phase II trial, PX-866 was well tolerated when in combination with cetuximab, but no apparent clinical benefits were observed. Idelalisib is usually used to cure lymphoma and leukemia rather than solid tumors. Akt inhibitors such as Perifosine, PIAs, and API-2 and mTOR inhibitors such as rapamycin, RAD-001, and CCI-779 have also been tested in clinical trials. PI3K-AKT-mTOR pathway is activated not only in CSCs but also in other cancer cells. Drugs targeting this pathway may not specifically target CSCs [82,121].

TGF-β is also a chemotherapeutic effective target for CSCs. Most TGF-β inhibitors directly target TGF-β receptors, such as LY2157299 (galunisertib), TEW-7197 (vactosertib), and anti-TGF-β monoclonal antibody SAR-439459. Galunisertib, an oral inhibitor of the TGF-β receptor type 1 kinase (ALK5), has completed several clinical trials with promising results [39,88,91,122]. In a phase I trial, Vactosertib, another ALK5 inhibitor, was tested on patients with advanced solid tumors. Vactosertib was well tolerated and had a therapeutic activity in patients with crizotinib-resistant ALK-positive advanced tumors.

### Therapies blocking the special niche of CSCs

CSCs inhibit CTL function through PD1-PDL1 crosstalk and CTLA-4-CD80/86 signaling and modulate macrophages through CD47, TGF-β, and CCL2 signaling. The PD1-PDL1 interaction is a well-known mechanism for CTL inhibition and immune evasion. Therefore, the antibodies of PD1-PDL1, including pembrolizumab, nivolumab, durvalumab, atezolizumab, avelumab, cimiplimab, and doltalimab, have achieved tremendous success in the treatment of different cancer types, For instance, Nivolumab is a human IgG4 monoclonal antibody that blocks PD-1. It shows promising efficacy and safety [73]. Pembrolizumab, an IgG4 isotype antibody intravenously injected, also performs well in terms of safety and efficiency. Pembrolizumab is well tolerated and associated with durable antitumor activity in multiple solid tumors [80], revealing its promising clinical activity for recurrent or metastatic tumors [90]. Avelumab, a human monoclonal antibody targeting PD-L1, significantly prolongs the overall survival of patients with urothelial cancer [84] and advanced renal cell carcinoma [70]. CTLA-4-CD80/86 is another well-known microenvironmental crosstalk for immune evasion. BMS-986218 has been conducted on patients with advanced lung carcinoma in phase I and II trials. Ipilimumab is an antibody against CTLA-4 and has been conducted on patients in combination with nivolumab in a series of trials [19,48,71,98,119].

On the other hand, therapies modulating the activities of macrophages have been emerging but are still on the way. For instance, Carlumab (CNTO888), an anti-CCL2 monoclonal antibody, has completed two clinical trials with no satisfactory results observed [6,81]. Carlumab alone or in combination with other chemotherapy regimens show well-tolerated properties, but no long-term suppression of serum CCL2 or significant tumor responses were observed.

## Conclusions and perspectives

In summary, CSCs represent a small subset of cancer cells within solid tumors. CSCs possess the unique ability for self-renewal and plasticity, allowing them to differentiate into different cell lineages. These distinctive behaviors are governed by specific intrinsic signaling pathways and their interactions with microenvironmental niches. These features distinguish CSCs from non-CSCs and render them resistant to conventional therapies designed for the bulk of tumor cells.

Efforts to eliminate CSCs have been developed based on these aforementioned unique properties. However, the plasticity of CSCs is often underestimated, leading to limited success in targeting them directly or via their microenvironmental niches. CSCs can evade therapies by differentiating into other unexpected cell lineages. Developing strategies to intervene in the plasticity of CSCs is a promising avenue for cancer treatment.

Another challenge in eradicating CSCs lies in the limited understanding of their interactions with the microenvironment. This is partly because many CSC studies use immunodeficient mouse models that lack an intact microenvironment, making it difficult to recapitulate the CSC niche comprehensively.

Finally, CSCs represent a small fraction of the overall cancer cell population, and their sparse representation makes it challenging to fully characterize their niche using single-cell RNA sequencing (scRNA-seq) or other techniques. Thus, a comprehensive understanding of the CSC niche remains a challenge in cancer research.

## Ethics, Consent to Participate, and Consent to Publish declarations

Not applicable.

## Funding

This work was supported by the grants 2021A1515110144, 2024B1515020020 and 2021A1515110051 from Guangdong Basic and Applied Basic Research Foundation, the grant 20220814161004001, RCYX20231211090317011 and JCYJ20220530113609020 from Science, Technology and Innovation Commission of Shenzhen Municipality, the grant 2021QN02Y875 from Department of Science and Technology of Guangdong Province, the grant 82273260, 82273079 and 81902787 from the National Natural Science Foundation of China, the grant GDRC202125 from Natural Science Foundation of Top Talent of SZTU, and the proposal “Teaching Practice of Medical Genetics Under Big Data Integration” from The Guangdong Province "New Medical Science" Teaching Reform Project.

## CRediT authorship contribution statement

**Jiali Xu:** Project administration, Writing – original draft. **Houde Zhang:** Project administration. **Zhihao Nie:** Project administration. **Wenyou He:** Project administration. **Yichao Zhao:** Writing – original draft. **Zhenhui Huang:** Project administration. **Lin Jia:** Supervision, Writing – review & editing. **Zhiye Du:** Writing – original draft. **Baotong Zhang:** Conceptualization, Supervision, Writing – original draft, Writing – review & editing. **Siyuan Xia:** Conceptualization, Supervision, Writing – original draft, Writing – review & editing.

## Declaration of competing interest

The authors declare no conflict of interest.
